# Hydrogen-rich water improves endurance by reducing skeletal muscle oxidative stress and inflammatory responses

**DOI:** 10.3389/fnut.2026.1722091

**Published:** 2026-01-20

**Authors:** Eika Mizuno, Tatsuhiro Sato, Kosuke Okada, Ikuru Miura, Junichi Shoda, Makoto Saito, Yutaro Mori, Sechang Oh, Tomonori Isobe

**Affiliations:** 1 Graduate School of Comprehensive Human Sciences, University of Tsukuba, Tsukuba, Japan; 2 Institute of Medicine, University of Tsukuba, Tsukuba, Japan; 3 Department of Pediatrics, University of Tsukuba Hospital, Tsukuba, Japan; 4 Faculty of Rehabilitation, R Professional University of Rehabilitation, Tsuchiura, Japan

**Keywords:** exercise endurance capacity, hydrogen-rich water, inflammation, oxidative Stress, skeletal muscle

## Abstract

**Introduction:**

Hydrogen-rich water (HRW) has been reported to reduce oxidative stress, suppress exercise fatigue, and enhance recovery. However, the molecular mechanisms of its effects on exercise capacity, especially during the early stages of adaptation in physically inactive individuals, remain unclear.

**Methods:**

Male 8-week-old C57BL/6 J mice were provided with purified water or HRW for 1, 2, 4, or 6 weeks. Their exercise endurance was assessed using treadmill running distance, and their levels of markers of oxidative stress, inflammation, and muscle damage were analyzed in skeletal muscle at baseline and after exercise at each time point.

**Results:**

Mice that consumed HRW for ≥P4 weeks ran significantly longer distances, showed less muscle fatigue, and had lower levels of markers of oxidative stress, inflammation, and muscle damage. Notably, antioxidant gene expression was not high, despite the lower level of oxidative stress, suggesting the possibility that HRW directly scavenges or suppresses reactive oxygen species, independently of antioxidant pathways.

**Discussion:**

HRW consumption alleviates oxidative stress and inflammation in skeletal muscle and improves exercise endurance. These findings suggest that the administration of HRW may represent a promising antioxidant strategy to support the initiation of and compliance with exercise programs by physically inactive individuals.

## 1 Introduction

Regular exercise is a fundamental lifestyle strategy that promotes metabolic health, strengthens the immune system, and reduces the risk of chronic disease (1). However, individuals who are new to regular exercise often struggle to maintain their exercise regimens because of the early onset of fatigue and inadequate recovery (2). Such early fatigue often leads to the premature discontinuation of exercise programs, which is a significant barrier to the establishment of good long-term exercise habits (3).

When the body is subjected to unfamiliar or exhausting exercise, there is an overproduction of reactive oxygen species (ROS), and particularly hydroxyl (OḤ) radicals (4), leading to oxidative stress (5). This oxidative stress can result in damage to lipids, proteins, and DNA, resulting in muscle damage, impaired skeletal muscle function, and inflammation (6). As a result, it becomes increasingly difficult for individuals, especially beginners, to engage in sustained physical activity, further discouraging the establishment of consistent exercise habits (3).

Although endogenous antioxidant defense mechanisms exist that neutralize ROS and maintain redox homeostasis, these mechanisms can be overwhelmed during the sudden surges in ROS production caused by unfamiliar or high-intensity exercise (5). Therefore, exogenous antioxidants have been evaluated as a means of mitigating exercise-induced oxidative stress. A number of dietary antioxidants, including vitamins C and E, polyphenols, flavonoids, and carotenoids, reduce oxidative damage, attenuate muscle fatigue, and improve exercise capacity (7, 8). These compounds act by scavenging free radicals or upregulating endogenous antioxidant defenses.

In our previous study, we demonstrated that sulforaphane, a compound found in cruciferous vegetables, activates the nuclear factor erythroid 2-related factor 2 (Nrf2) pathway, a critical regulator of antioxidant defenses. The activation of Nrf2 increases the expression of endogenous antioxidant enzymes and protective proteins, thereby enhancing the ability of cells to detoxify ROS and maintain redox balance. In animal models, sulforaphane supplementation reduces oxidative stress and improves exercise endurance (9).

More recently, molecular hydrogen (H_2_) has emerged as a novel antioxidant with unique properties (10). In addition to activating the Nrf2 pathway (11), H_2_ directly neutralizes OḤ radicals, converting them to harmless water without interfering with other ROS that are involved in essential cellular signaling processes (12). Unlike many pharmacological antioxidants, whose efficacy may be limited by poor intracellular penetration or dependence on transporter mechanisms, H_2_, owing to its small size and nonpolar nature, can rapidly and passively diffuse into cells and organelles, including mitochondria, which are the primary sites of ROS production during exercise (12–14). These properties suggest that H_2_ may directly mitigate oxidative stress, reduce muscle damage, and support sustained exercise performance (15). This is particularly relevant for individuals who are unaccustomed to regular exercise because improvements in the management of oxidative stress and fatigue may facilitate the development of consistent exercise habits. Indeed, previous studies have shown that molecular hydrogen supplementation can suppress fatigue and enhance recovery during and after physical exertion (16, 17).

Despite these promising findings, the effects of H_2_ on exercise capacity and the precise mechanisms underlying these effects remain poorly characterized (18). Therefore, we investigated the effects of hydrogen-rich water (HRW) supplementation on the exercise capacity, oxidative stress markers, and muscle damage indicators of previously untrained mice. We specifically focused on the antioxidant effects of H_2_, including Nrf2 activation and direct ROS neutralization. We hypothesized that H_2_ supplementation would improve exercise performance by reducing ROS-induced cellular damage and supporting endogenous antioxidant defenses.

## 2 Materials and methods

### 2.1 Animals and treatments

**Figure 1:**
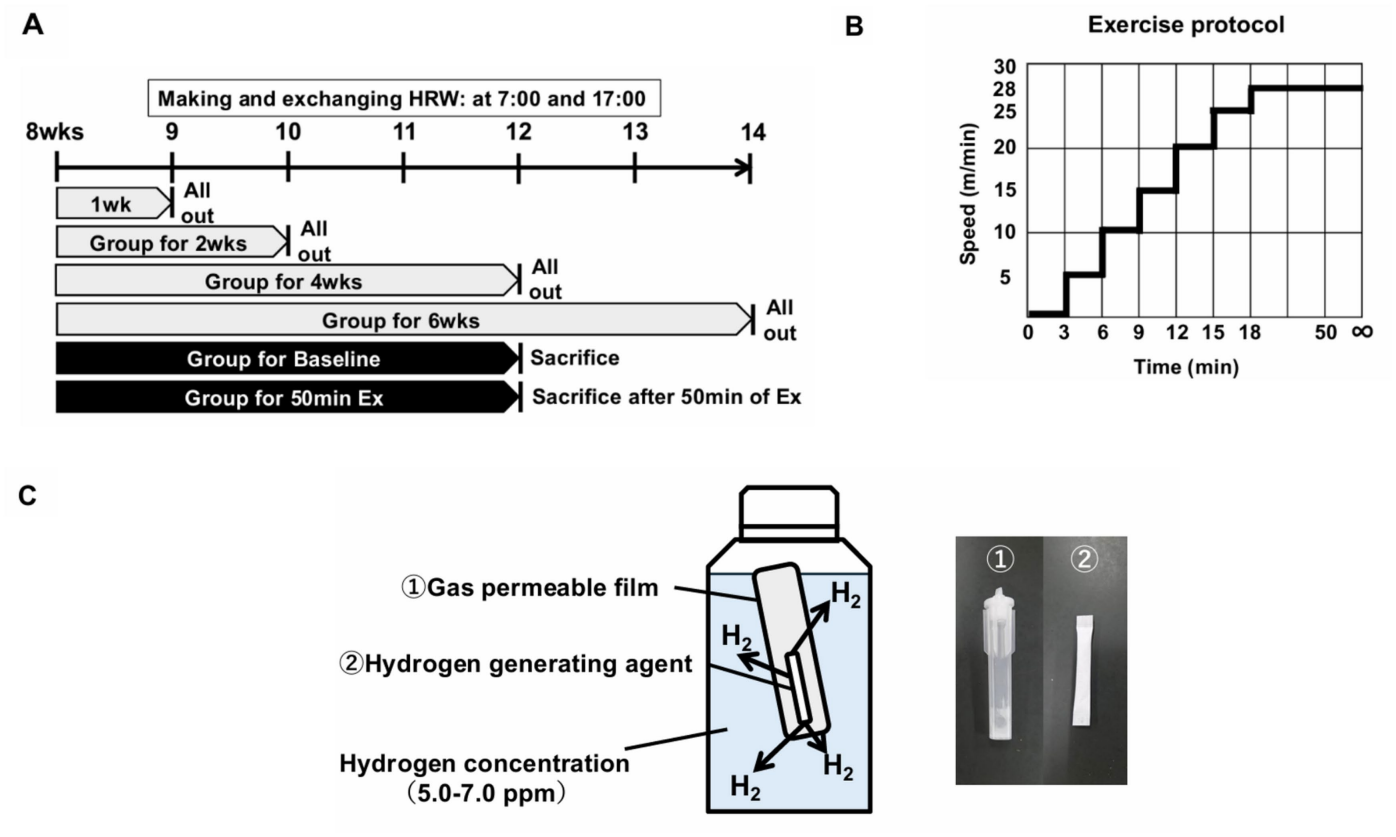
Experimental design and preparation of hydrogen-rich water (HRW). **(A)** Experimental timeline and description of the groups based on the duration of water intake (1, 2, 4, or 6 weeks), with or without exercise loading prior to tissue collection. **(B)** Treadmill exercise protocol. Running speed was increased stepwise to 28 m/min and maintained thereafter. The exercise was either stopped at 50 min (*50 min Ex*) or continued until exhaustion (*All-out*). **(C)** Preparation of HRW: a hydrogen-generating agent sealed with a gas-permeable film to maintain a stable hydrogen concentration.

All animal procedures were conducted in accordance with international guidelines for the care and use of laboratory animals and approved by the Institutional Animal Care and Use Committee of the University of Tsukuba (Ibaraki, Japan). Eight-week-old male C57BL/6 J mice were housed in colony cages under a 12 h light /12 h dark cycle at controlled temperature (22.5 ± 1.4 °C) and humidity (55.6% ± 4.0%), with free access to a standard pelleted diet and water. The mice were randomly assigned to one of six experimental groups (Figure 1A). Four of the groups were administered either purified water (PW) or HRW for 1, 2, 4, or 6 weeks, then they were subjected to a treadmill running test to evaluate their exercise capacity (Figure 1B). Each time point was evaluated independently. The final group sizes were 1 week – PW, *n* = 6; HRW, *n* = 6; 2 weeks – PW, *n* = 4; HRW, *n* = 5; 4 weeks – PW, *n* = 10; HRW, *n* = 10; and 6 weeks – PW, *n* = 5; HRW, *n* = 5. These variations in sample size reflected experimental attrition and the inclusion of an additional reproducibility experiment at the 4-week time point. The remaining two groups received either PW or HRW for 4 weeks and were then sacrificed either without performing exercise or immediately after a single 50-min treadmill running session. These groups permitted the assessment of the physiological and molecular effects of HRW both at baseline and in response to substantial acute exercise. Exhaustion was defined as failure to resume forward running after falling over, or remaining on the shock grid for more than 5 consecutive seconds (9). Behavioral and physiological assessments were performed by assessors blinded to the group allocation, whereas biochemical analyses were conducted without blinding.

### 2.2 Preparation of hydrogen-rich water

HRW was prepared using a 7Water Premium device (MiZ Co., Ltd., Kamakura, Japan) according to the manufacturer’s protocol (Figure 1C). To minimize hydrogen loss, a specially coated PET bottle and a custom-designed drinking container for research use, supplied by MiZ, were used throughout the preparation and administration processes. According to the manufacturer’s instructions, the generated HRW underwent a 24-h stabilization process during the initial preparation. Preliminary verification confirmed that these containers were able to maintain hydrogen concentrations at >5 ppm for up to 14 h. Based on this, HRW was provided to the animals in fresh water twice daily at 07:00 and 17:00 to ensure consistent hydrogen exposure.

### 2.3 Sample collection

The mice were sacrificed by excess isoflurane inhalation at one of two experimental time points: either immediately after 4 weeks of consumption of PW or HRW without exercise or immediately following a 50-min treadmill running session subsequent to the same 4-week water consumption period. At sacrifice, soleus, plantaris, gastrocnemius, tibialis cranialis, extensor digitorum longus, and quadriceps muscles from both hindlimbs were rapidly dissected. These muscles were selected on the basis of their physiological characteristics and the amount of tissue required for the respective analyses. Blood samples were collected from the abdominal aorta and centrifuged at ~660 × *g* for 20 min at 4 °C to isolate serum. All collected muscle and serum samples were immediately snap-frozen in liquid nitrogen and stored at −80 °C until further analysis.

### 2.4 Grip strength

The grip strength of the mice was measured at 8, 9, 10, 11, and 12 weeks of age using a grip strength meter for mice (MK-380K, Muromachi Kikai, Tokyo, Japan). The measurements were made by pulling the mouse’s tail backward with its limbs grasping the meter. The measurements were repeated five times and the mean values were recorded. All the measurements were performed by the same investigator.

### 2.5 Histochemical analysis

Gastrocnemius muscles were embedded in tragacanth gum (Fujifilm Wako, Osaka, Japan, Cat# 206–00591), rapidly frozen in isopentane (Nacalai Tesque Inc., Kyoto, Japan Cat# 26404–75), cooled with liquid nitrogen, and cryosectioned at an 8 μm thickness. The sections obtained were mounted on glass slides. For hematoxylin and eosin (HE) staining, the sections were treated with Mayer’s hematoxylin (ProBiotek, San Nicolás de los Garza, Mexico, Cat# 131–09665) for 7 min, rinsed under warm tap water (~50 °C) for 3 min, and then stained with eosin Y (Fujifilm Wako, Cat# 051–06515) for 50 s. They were dehydrated in 95 and 100% ethanol (2 × each, 5 s), cleared in xylene (3×, 5 min), and mounted with Distyrene–Plasticizer–Xylene.

(Sigma-Aldrich, St. Louis, MO, USA). Images were captured using a BZ-X810 microscope (Keyence, Osaka, Japan). For immunofluorescence, cryosections were fixed in cold acetone (−20 °C), air-dried, and blocked with 5% goat serum/1% bovine serum albumin in PBS and mouse on mouse reagent (Vector Laboratories, Newark, CA, USA). Primary antibodies against myosin heavy chain isoforms were applied overnight at 4 °C (type I (BA-D5), IIa (SC-71), IIx (6H1), or IIb (BF-F3) (all from DSHB, Iowa City, IA, USA)). After washing, Alexa Fluor™-conjugated secondary antibodies (Thermo Fisher, Waltham, MA, USA) were applied (IgG2b–350 (A-21140), IgG1–555 (A-21127), or IgM–488 (A-21042)). The slides were mounted with Vectashield Vibrance (Vector) and examined using the BZ-X810 microscope.

### 2.6 Indirect calorimetry

Oxygen consumption (VO₂) was measured during a 50-min treadmill running session using an indirect calorimeter (Muromachi Kikai, Tokyo, Japan) at the Laboratory Animal Resource Center, University of Tsukuba. Measurements were made continuously throughout the exercise period, with the VO₂ being recorded every 3 min. The air flow rate was maintained at 0.6 L/min during the measurement period.

### 2.7 Antibodies and immunoblot analysis

For western blot analysis, cell lysates were prepared from the tibialis cranialis muscle, their protein concentrations were quantified and normalized, and then the lysates were subjected to SDS–PAGE, after which the proteins were transferred to nitrocellulose membranes (Bio-Rad, Hercules, CA, USA). Membranes were blocked for 1 h at room temperature using Blocking One-P (Nacalai Tesque Inc.), followed by overnight incubation at 4 °C with the following primary antibodies: phospho-NF-κB p65 (1:1,000, Cell Signaling Technology, Danvers, MA, USA), NF-κB (1:1,000, Cell Signaling Technology), Nrf2 (1:500, Proteintech, Rosemont, IL, USA), and *β*-actin (1:5,000, Sigma-Aldrich). After washing, the membranes were incubated with appropriate horseradish peroxidase-conjugated secondary antibodies for 2 h at room temperature. Signals were visualized using Chemi-Lumi One Super (Nacalai Tesque Inc.) and detected using the ChemiDoc XRS + imaging system (Bio-Rad). Image acquisition and densitometric analysis were performed using Image Lab software (Bio-Rad).

### 2.8 Real-time quantitative polymerase chain reaction

Quantitative real-time PCR was performed to analyze gene expression using the CFX384 Touch Real-Time PCR Detection System (Bio-Rad). Two microliters of DNA (diluted 1:10) were added to Fast SYBR Green Master Mix (Applied Biosystems, Santa Ana, CA, USA) in a total volume of 20 μL. The PCR cycling conditions comprised an initial enzyme activation at 95 °C for 20 s, followed by 40 cycles of denaturation at 95 °C for 1 s and annealing at 60 °C for 20 s. Fluorescence was measured at the end of each cycle, and melting curve analysis was performed from 65 to 95 °C with 0.1 °C/s increments. Each sample was analyzed in triplicate with appropriate negative controls. Target gene expression levels were calculated using the delta-Ct method and normalized to that of *Gapdh*. The following primers (Fasmac Co., Ltd., Atsugi, Japan) were used: *Hmox1*: F: 5′-CCTCACTGGCAGGAAATCATC-3′, R: 5′-ATACATTTCGCAGAGGTGCTCC-3′; *Nqo1*: F: 5′-GGGTCGTCTTGGCAACCA-3′, R: 5′-AATGCTAGGAGGGAGTTGTAGAC-3′; *Cat*: F: 5′-GGTCACCCACGATATATCACCAGATAC-3′; R: 5′-ACTGTGTCAAGCACTGGGAGC-3′; and *Gapdh*: F: 5′-GTCTTCACCACCATGGAGAAGGCT-3′, R: 5′-ACTTGCCCTTCGAGTGACCGTAC-3′.

### 2.9 Biochemical analysis

The serum activities of lactate dehydrogenase (LDH) and creatine kinase (CK) were measured using the transferable methods of the Japan Society of Clinical Chemistry. The thiobarbituric acid-reactive substance (TBARS) concentrations in tibialis cranialis muscles were quantified using a commercial ELISA kit (Cayman Chemical, Ann Arbor, MI, USA). The reduced (GSH) and oxidized (GSSG) glutathione levels in gastrocnemius muscle were determined using a kit from Dojindo Molecular Technologies (Kumamoto, Japan). The superoxide dismutase (SOD) activity in gastrocnemius muscle was measured using a SOD Assay Kit-WST (Dojindo Molecular Technologies). Serum concentrations of 8-hydroxy-2′-deoxyguanosine (8-OHdG) were measured using an ELISA kit (Biovision, #K4160-100, Milpitas, CA, USA).

### 2.10 Statistics

Statistical analyses were performed using SPSS Statistics for Mac, version 29 (IBM Corp., Armonk, NY, USA). Data are presented as mean ± standard error of the mean. The body-weight, grip-strength, and VO_2_ time-course measurements, which were made repeatedly in the same animals, were analyzed using linear mixed-effects (LMM) models. For the other single-time-point comparisons, the unpaired *t*-test was used to compare the PW and HRW groups. A *p-*value of < 0.05 was considered to indicate statistical significance.

## 3 Results

### 3.1 Baseline assessment

**Figure 2:**
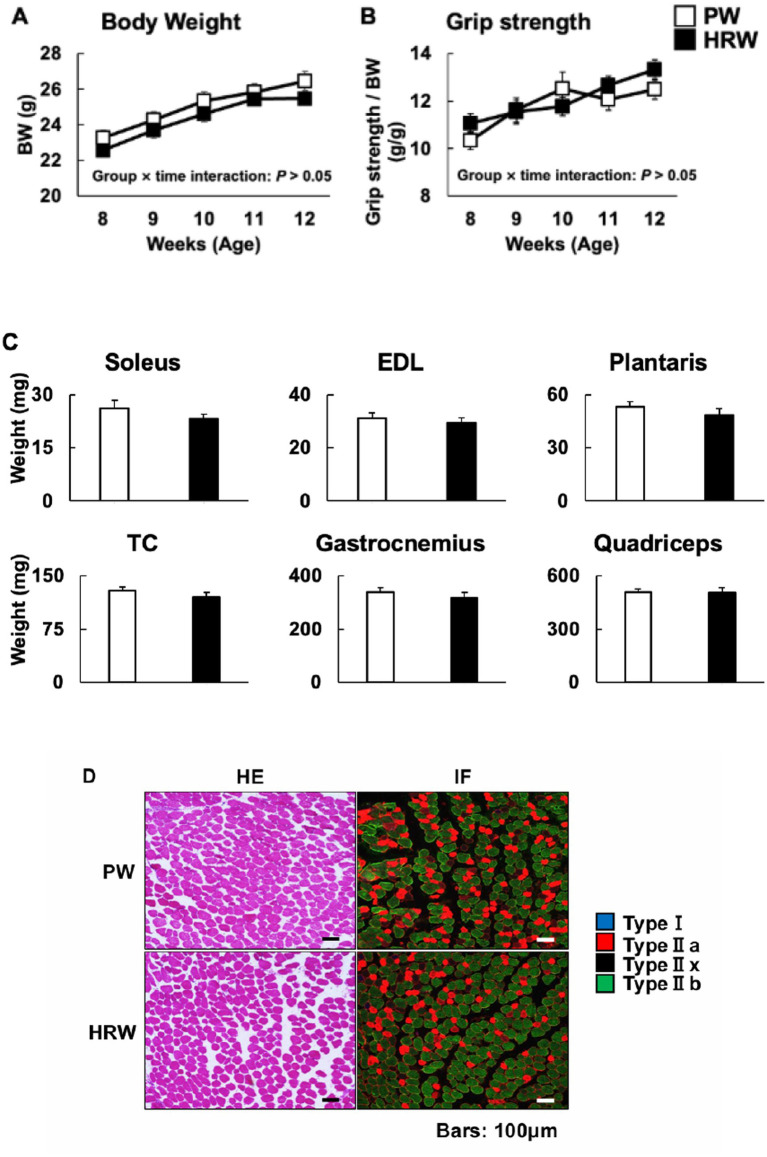
Baseline morphological and physiological characteristics of the mice following HRW intake. **(A)** Body weight (g) of the mice consuming purified water (PW) or hydrogen-rich water (HRW) (PW: *n* = 12 per group, HRW: *n* = 14 per group). **(B)** Forelimb grip strength (g/g), measured weekly during the intervention period (PW: *n* = 12 per group, HRW: *n* = 14 per group). **(C)** Wet masses of six hindlimb skeletal muscles (soleus, extensor digitorum longus (EDL), plantaris, tibialis cranialis (TC), gastrocnemius, and quadriceps femoris) (PW: *n* = 12 per group, HRW: *n* = 14 per group). **(D)** Representative images of gastrocnemius muscle cross-sections that were hematoxylin and eosin (HE)-stained or fluorescence immunostained (IF) for myosin heavy chain isoforms (*n* = 3 per group).

The body weights of the mice increased progressively from week 8 to week 12 in both groups. LMM analysis showed a significant main effect of time (*p* < 0.001), but neither the group effect (*p* > 0.05) nor the group × time interaction (*p* > 0.05) was significant, indicating that HRW consumption did not influence the longitudinal change in body weight (Figure 2A). The forelimb grip strength of the mice progressively increased from week 8 to week 12 in both groups (Figure 2B). LMM analysis revealed a significant main effect of time (*p* < 0.001), whereas neither the group effect nor the group × time interaction was significant (both *p* > 0.05). These results indicate that HRW consumption did not influence the longitudinal change in grip strength. The weights of the dissected skeletal muscles (the soleus, extensor digitorum longus, plantaris, tibialis cranialis, gastrocnemius, and quadriceps femoris) also did not differ significantly between the groups (Figure 2C). Furthermore, HE staining and immunofluorescent fiber-type staining of the gastrocnemius muscle revealed no notable differences in muscle morphology or fiber-type distribution (Figure 2D). Thus, continuous HRW consumption did not alter the physical or histological characteristics of the skeletal muscle of the mice.

### 3.2 Exercise endurance capacity and energy metabolism parameters

**Figure 3:**
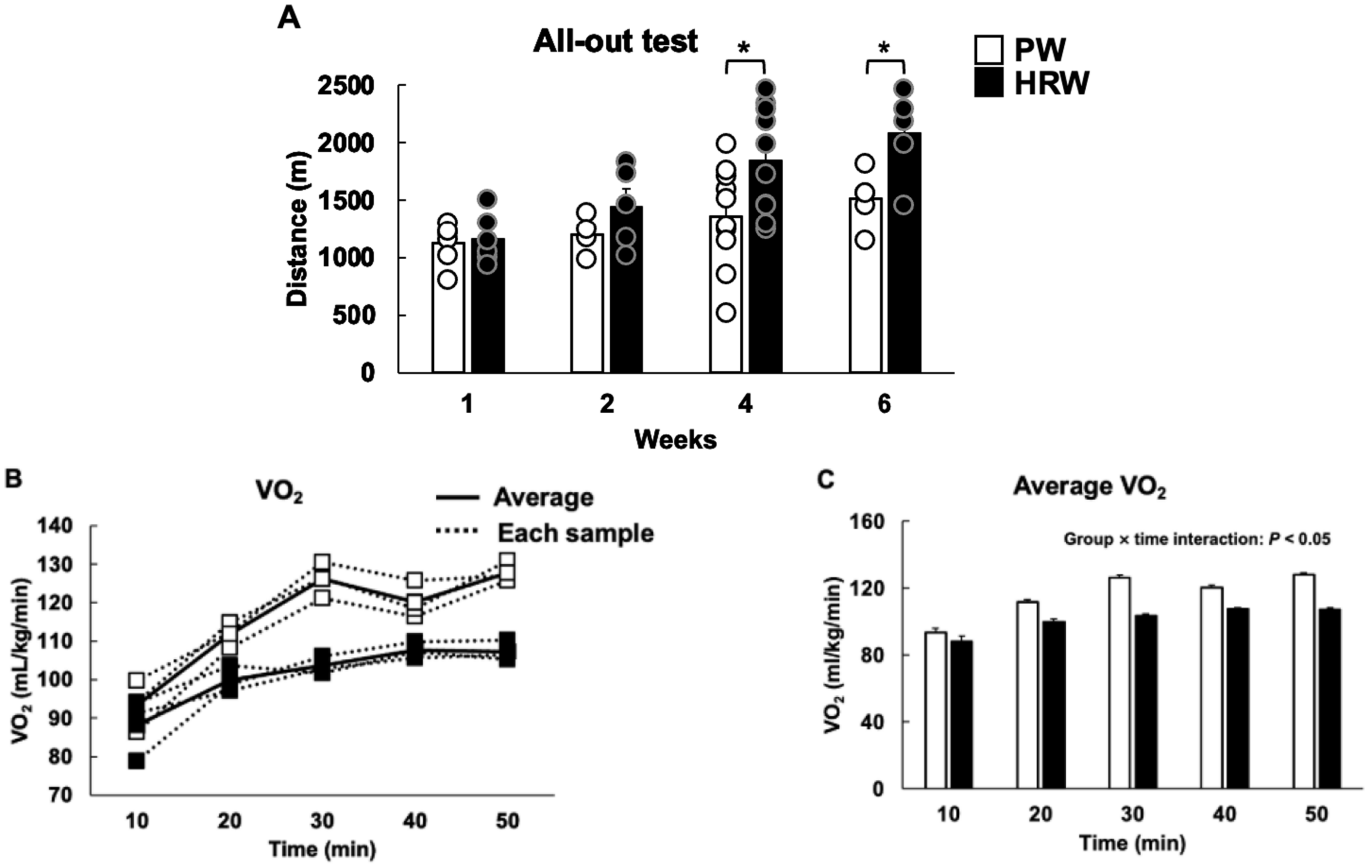
Effects of HRW consumption on the endurance and VO_2_ of the mice. **(A)** Running distance during a treadmill test after 1, 2, 4, or 6 weeks of PW or HRW intake. Each time point was evaluated independently. The group sizes at each time point were as follows: 1 week – PW, *n* = 6; HRW, *n* = 6; 2 weeks – PW, *n* = 4; HRW, *n* = 5; 4 weeks – PW, *n* = 10; HRW, *n* = 10; 6 weeks – PW, *n* = 5; HRW, *n* = 5. **(B)** Time course of VO_2_ during 50 min of treadmill exercise following 4 weeks of PW or HRW consumption (*n* = 4 per group). **(C)** Mean VO₂ during the 50-min exercise session (*n* = 4 per group). The data in panel C were analyzed using LMM analysis *p < 0.05.

The running distance of the mice in the PW and HRW groups during the treadmill test did not significantly differ after 1 or 2 weeks of water consumption. However, after 4 and 6 weeks, the HRW mice ran significantly longer distances than the PW mice (Figure 3A). VO_2_ increased progressively over the 50-min running session in both groups (Figures 3B, C). LMM analysis revealed a significant main effect of time (*p* < 0.001), consistent with the expected rise in VO_2_ during sustained exercise. Although the overall group effect was not significant (*p* > 0.05), the group × time interaction was significant (*p* < 0.05), indicating that the trajectory of the increase in VO_2_ differed between the groups. Specifically, the mice in the HRW group exhibited a smaller increase in VO_2_ than PW mice during prolonged running, suggesting lower metabolic demand and a delay in the development of fatigue.

### 3.3 Nrf2 target genes

**Figure 4:**
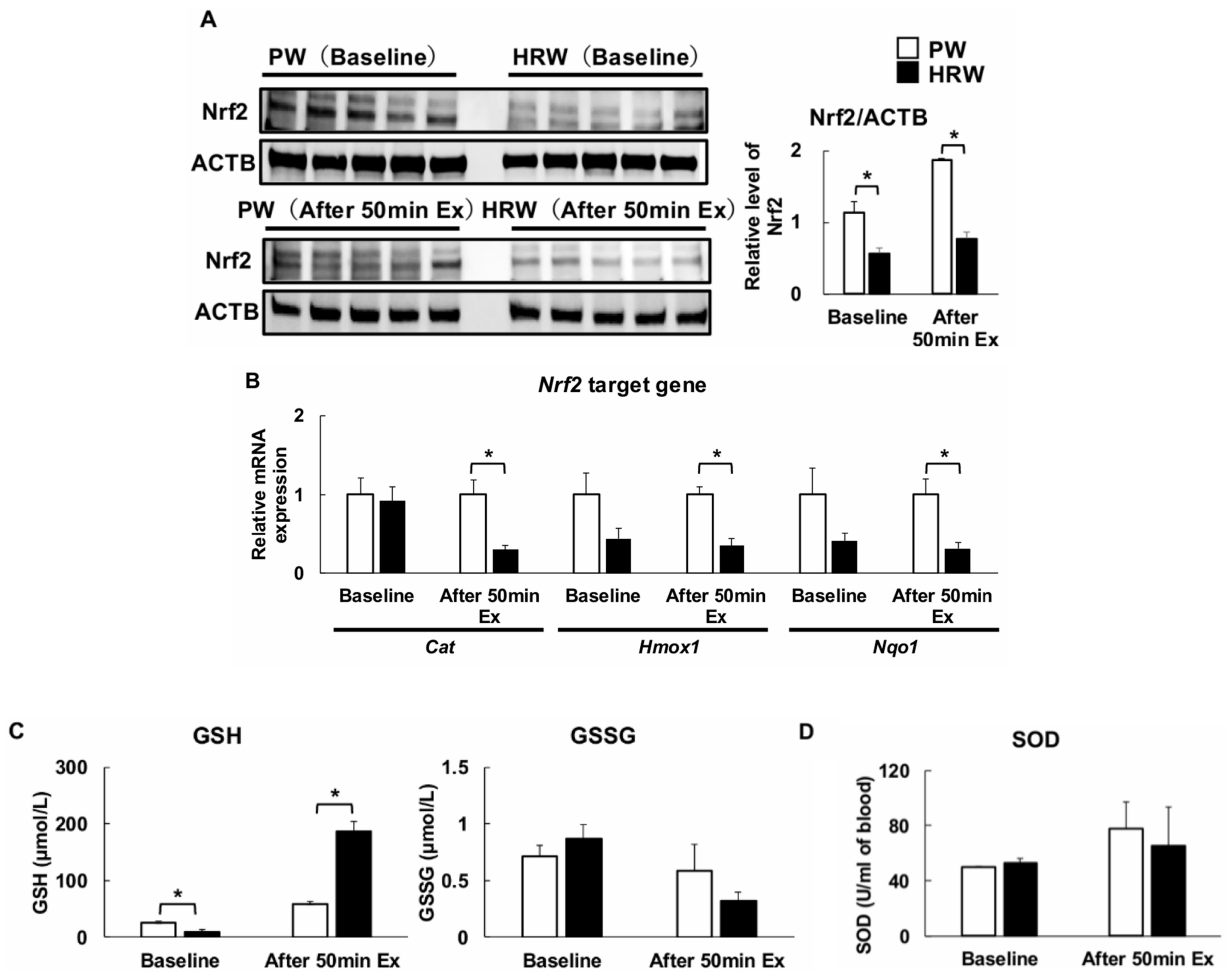
Effects of HRW consumption on Nrf2 signaling in and the redox status of the skeletal muscle of the mice. **(A)** Immunoblot analysis of Nrf2 protein expression in the tibialis cranialis muscle at baseline and after 50 min of treadmill exercise (*n* = 5 per group). **(B)** mRNA expression of Nrf2 target genes (*Cat*, *Hmox1*, *Nqo1*) in the tibialis cranialis muscle, evaluated using qPCR, at baseline and after 50 min of exercise (*n* = 5 per group). **(C)** Reduced (GSH) and oxidized (GSSG) glutathione levels in the gastrocnemius muscle at baseline and after 50 min of exercise (*n* = 4 per group). **(D)** Superoxide dismutase (SOD) activity in the gastrocnemius muscle at baseline and after 50 min of exercise (*n* = 4 per group). *p < 0.05 *vs*. PW group.

Immunoblot analysis of the tibialis cranialis muscle showed that Nrf2 protein expression was significantly lower in the HRW group than in the PW group, both at baseline and after 50 min of exercise (Figure 4A). qPCR analysis revealed no significant differences in the expression of *Cat*, *Hmox1*, or *Nqo1* between the PW and HRW groups at baseline. However, after 50 min of exercise, the expression of these antioxidant genes was significantly lower in the HRW group than in the PW group (Figure 4B). In the gastrocnemius muscle, the baseline GSH levels were also significantly lower in the HRW group than in the PW group (Figure 4C), but after exercise, the GSH levels were significantly higher in the HRW group than in the PW group. There were no significant differences in the SOD activity in the gastrocnemius muscle between the groups, either at baseline or after exercise (Figure 4D).

### 3.4 Biomarkers of oxidative stress and muscle damage

**Figure 5:**
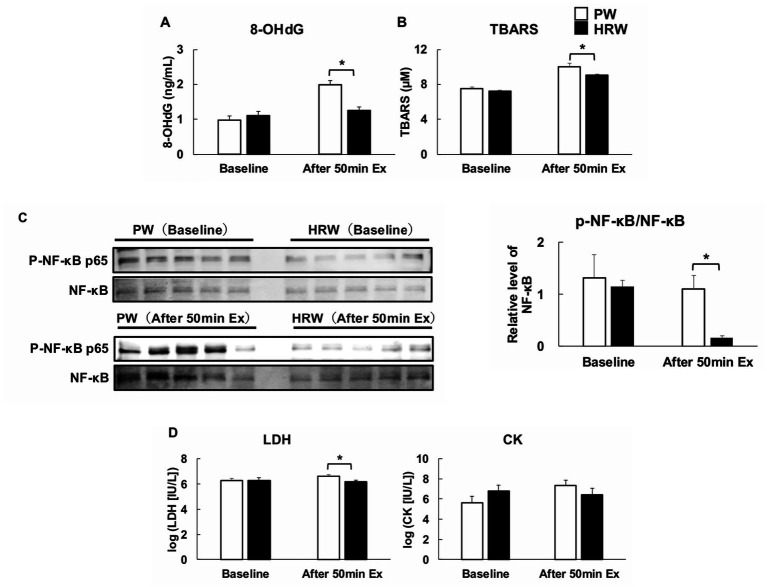
Effects of HRW on oxidative stress, inflammation, and muscle damage after endurance exercise in the mice. **(A)** Serum 8-hydroxy-2′-deoxyguanosine (8-OHdG) levels at baseline and after 50 min of treadmill exercise (*n* = 5 per group). **(B)** Thiobarbituric acid-reactive substance (TBARS) content of the tibialis cranialis muscle at baseline and after exercise (*n* = 4 per group). **(C)** Immunoblot analysis of NF-κB and phosphorylated NF-κB p65 in the tibialis cranialis muscle at baseline and after 50 min of exercise (*n* = 5 per group). **(D)** Serum activities of lactate dehydrogenase (LDH) and creatine kinase (CK) at baseline and after 50 min of exercise (*n* = 5 per group). *p < 0.05 *vs*. PW group.

The serum concentrations of 8-OHdG did not differ at baseline, but were significantly lower in the HRW group than in the PW group after 50 min of exercise (Figure 5A). Similarly, the TBARS content of the tibialis cranialis muscle was significantly lower in the HRW group after exercise, but not at baseline (Figure 5B). Immunoblot analysis of the tibialis cranialis muscle revealed no difference in NF-κB expression at baseline, but the expression was significantly lower in the HRW group than in the PW group following 50 min of exercise (Figure 5C). The serum LDH activities of the groups did not differ at baseline, but were significantly lower in the HRW group after exercise. In contrast, the serum CK activities did not differ between the groups in either situation (Figure 5D).

## 4 Discussion

H₂ readily diffuses into various cellular compartments because of its small size and nonpolar nature (19, 20). This diffusion has the potential to permit the scavenging of ROS or inhibit their production in excess (13, 21). The selective antioxidant activity of H₂ has been demonstrated to alleviate oxidative damage under a variety of conditions, including in the presence of metabolic disorders, cardiovascular diseases, and exercise-induced stress (22, 23). In the present study, we aimed to determine whether prolonged consumption of HRW, through its antioxidant effects, including Nrf2 pathway activation and direct ROS neutralization, would improve exercise endurance, attenuate oxidative stress, and reduce muscle damage in previously untrained mice.

The key findings of the study were as follows. First, ≥4 weeks of consumption of HRW caused a significant increase in running distance, whereas no improvement was achieved following only 1 or 2 weeks. Second, HRW consumption suppressed the increase in VO_2_ during 50 min of exercise, implying that there was less metabolic stress and that the mice may have experienced less fatigue at the same exercise intensity. Third, reductions in the activity of a marker of muscle damage (LDH) and the levels of markers of oxidative stress (8-OHdG and TBARS) and inflammation (NF-κB phosphorylation) were identified following exercise. Although the serum CK activity did not show a statistically significant difference between groups, the HRW mice tended to have lower CK activities, suggestive of a small but biologically meaningful reduction in muscle stress. Notably, these improvements occurred even though Nrf2 expression, that of downstream antioxidant genes (*Hmox1*, *Cat*, and *Nqo1*), and the circulating concentrations of GSH and SOD were not increased; indeed, some of these were lower than those of the control group after exercise. These findings suggest that a sustained reduction in ROS and the control of inflammatory responses result in an increase in exercise endurance and delay fatigue, without the need for Nrf2 activation or the upregulation of the associated antioxidant enzymes.

The identified reductions in muscle damage, oxidative stress, and inflammation following HRW supplementation can be attributed to the multifaceted antioxidant and anti-inflammatory effects of H₂ (24). By regulating ROS at the cellular level, hydrogen is likely to prevent excessive lipid peroxidation and protein oxidation, which compromise membrane integrity and cellular components in muscle (24–26). In particular, H₂ can directly neutralize OḤ radicals (H_2_ + 2OH → 2H_2_O), the most reactive and damaging type of ROS, thereby providing an immediate protective effect against severe biomolecular damage (10, 27, 28). The attenuation of ROS reduces the activation of the cascade, leading to the release of damage-associated molecular patterns and subsequent inflammatory pathway activation (29, 30). Consequently, the principal transcription factors, such as NF-κB, remain relatively inactive, which reduces proinflammatory cytokine production and establishes a stable internal environment conducive to the preservation of muscle fiber function (14, 31, 32). Moreover, this decrease in oxidative damage may preserve mitochondrial integrity and increase the efficiency of energy production, thereby curbing secondary ROS formation and interrupting the cycle of oxidative and inflammatory stress (14, 20, 33, 34). In this way, the ability of H₂ to sustain redox homeostasis serves to protect against the structural and functional disruption of muscle tissue and reduce fatigue, leading to an improvement in exercise endurance.

It should be noted that an unexpected finding was that after 4 weeks of HRW consumption, the Nrf2 levels were significantly lower than those of the control mice. In addition, the baseline circulating levels of GSH, which are regulated in part by the Nrf2-driven transcription of glutathione biosynthetic enzymes, were also lower than those of the control group. Moreover, following 50 min of exercise, not only were the Nrf2 levels lower, but the Nrf2-related antioxidant genes (*Hmox1*, *Cat*, and *Nqo1*) were expressed at lower levels than in control mice. In addition, the baseline activities of SOD, another critical antioxidant enzyme that is regulated in part by Nrf2 signaling, did not differ between the HRW and control groups, and this is also consistent with HRW consumption not stimulating conventional Nrf2-driven antioxidant enzyme pathways. Intriguingly, the baseline levels of reduced GSH were significantly lower in the HRW group, implying that the early neutralization or suppression of ROS by hydrogen reduced the baseline requirement for GSH. However, immediately following exercise, GSH levels were significantly higher in the HRW group than in the control mice, suggesting that the direct and rapid neutralization of ROS by hydrogen during physical exertion protected cellular GSH pools from oxidative depletion or facilitated their regeneration.

Although some previous studies have demonstrated that H₂ increases antioxidant enzyme expression by increasing Nrf2 activation (35, 36), the present data suggest that the suppression of ROS accumulation alone can maintain a stable antioxidant environment without the need for Nrf2-dependent compensatory mechanisms. In other words, H₂ may serve as a strategic antioxidant, modulating its mode of action in accordance with the conditions, rather than its effects being mediated through a single signaling pathway (37–39). This indicates that antioxidant defenses may be more flexible and contextdependent than previously assumed (15, 40). Rather than implying a single defined mechanism, these observations may reflect several possible redox regulatory processes. By regulating ROS at an earlier stage, H_2_ may reduce the necessity for Nrf2-driven antioxidant enzyme upregulation (24, 28, 41), although lower mitochondrial ROS production, the involvement of transcription factors other than Nrf2, or shifts in the basal redox set-point may also contribute to these patterns. The significantly lower Nrf2 expression in the HRW group, in conjunction with the lower expression of *Hmox1*, *Cat*, and *Nqo1* following exercise and the stable SOD activity irrespective of exercise status, may therefore suggest a potential, rather than a definitive, form of adaptation in which the body, being accustomed to persistently low ROS levels, no longer requires Nrf2 activation. This should be regarded as a plausible hypothesis, whereby early ROS control by hydrogen reduces the necessity for additional Nrf2-mediated defenses, and activation of antioxidant enzymes such as SOD, although further studies will be required to determine the precise mechanisms involved. This strategic antioxidant approach is further exemplified by the preservation and post-exercise increase in GSH levels that were identified following HRW supplementation. This flexibility distinguishes H₂ from other antioxidants and is the basis for its safety and efficacy.

A comparison with previous studies of H₂ and exercise demonstrated that the majority of previous studies evaluated acute H₂ supplementation or inhalation, and demonstrated improvements in various recovery-related indices, such as lactate clearance, or the rapid normalization of oxidative status (18, 42, 43). Some studies have focused on Nrf2-mediated defenses or mitochondrial protection (34, 36), while others have focused on the direct radical-scavenging properties of H₂ (10, 37). However, these investigations frequently involved brief intervention periods or focused on specific biomarkers, which limited their ability to determine whether these protective effects could be sustained over time and result in an improvement in exercise endurance capacity. In contrast, the present study has demonstrated that the cumulative antioxidant and anti-inflammatory effects of HRW can, over time, result in meaningful improvements in exercise capacity. It is reasonable to think that the control of ROS and inflammation, even in the absence of Nrf2 activation, could contribute to a more favorable physiological environment that could support greater endurance. However, these findings reflect physiological adaptations in mice, and any implications for human exercise remain speculative, because behavioral outcomes were not assessed in the present study.

It is important to acknowledge that the present study has some limitations. First, we did not characterize the metabolic adaptations that occurred, such as changes in energy substrate utilization, mitochondrial efficiency, and nutrient processing. These aspects will be the focus of subsequent research. Second, the conclusions drawn on the basis of the use of the particular animal model and exercise modality employed in the study may not be directly applicable to other species, exercise types, or human populations, and caution is therefore required when interpreting the translational relevance of the findings. In addition, the tissue and blood hydrogen concentrations were not directly measured in the present study, which represents an important limitation. Future studies that incorporate direct hydrogen measurements will be necessary to evaluate hydrogen availability at the tissue level. In addition, because only male mice were used, potential sex differences could not be evaluated, and future studies that include both sexes will be needed to determine whether responses to HRW differ in the two sexes. Furthermore, the mechanisms proposed in the present study should be regarded as plausible hypotheses rather than definitive conclusions, and additional work will be required to verify the specific pathways involved. Also, several molecular analyses were conducted using relatively small sample sizes, which should be considered when interpreting the molecular findings. Finally, it should be noted that the effects of HRW consumption may be influenced by a number of variables, including age, training status, and lifestyle. Further research is required to determine the optimal duration of consumption, dosage, and concentration, as well as to evaluate potential synergies with other nutritional or training strategies. Rigorously controlled human trials will be essential to determine whether the physiological effects identified in the mice can be reproduced in humans.

## 5 Conclusion

The results of the present study demonstrate that long-term HRW consumption reduces the adverse effects of oxidative stress and inflammation following exercise, attenuates muscle damage, and improves exercise capacity. These findings indicate that hydrogen supplementation does not just have short-term protective effects, but can also serve as a versatile antioxidant strategy with tangible benefits to performance. The capacity of H₂ to preserve stable antioxidant conditions without predominantly relying on Nrf2 activation highlights its adaptable and responsive mechanisms of action.

## Data Availability

The raw data supporting the conclusions of this article will be made available by the authors, without undue reservation.

## Glossary

8-OHdG: 8-hydroxy-2′-deoxyguanosine
CK: Creatine kinase
*Gapdh*: Glyceraldehyde-3-phosphate dehydrogenase
GSH: Reduced glutathione
GSSG: Oxidized glutathione
HE: Hematoxylin and eosin
*Hmox1*: Heme oxygenase-1
HRW: Hydrogen-rich water
IgG: Immunoglobulin G
IgM: Immunoglobulin M
LDH: Lactate dehydrogenase
LMM: Linear mixed-effects model
NF-κB: Nuclear factor-κB
*Nqo1*: NAD(P)H quinone oxidoreductase 1
Nrf2: Nuclear factor erythroid 2-related factor 2
PW: Purified water
ROS: Reactive oxygen species
SOD: Superoxide dismutase
TBARS: Thiobarbituric acid-reactive substances
VO₂: Volume of oxygen consumed
